# Supplementary material for: Ultraslow isomerization in photoexcited gas-phase carbon cluster C10-
Source: Nat Commun. 2018 Mar 2;9:912. doi: 10.1038/s41467-018-03197-w (PMC5834543; doi:10.1038/s41467-018-03197-w)
Supplement: Supplementary file 1 — Supplementary Information [file 41467_2018_3197_MOESM1_ESM.pdf]

Ultraslow isomerization in photoexcited gas-phase carbon cluster C_{10}^-

Saha *et al.*

Supplementary Note 1: Neutralization of C_{10}^- upon photoexcitation at different trapping times

The internal energy of excited C_{10}^- trapped in the EIBT decreases as the trapping time increases. Thus when the ions are photoexcited at shorter trapping times, their total internal energy (initial internal energy + photon energy) is higher than at longer trapping times. The neutral counts from C_{10}^- as a function of time after the laser interaction at different trapping times are shown in Supplementary Figure 1 for photoexcitation energy of 2.85 eV.

Since the total internal energy of C_{10}^- is higher when photoexcited after shorter trapping times, the neutralization rate is faster, because the rates of the relaxation mechanisms governing the neutralization rate (which in our case is mainly isomerization) increase with energy. As time progresses, the cluster cools down by radiative relaxation mechanisms and consequently the internal energy decreases, resulting in a slower neutralization rate when C_{10}^- is photoexcited. We calculated the neutralization rates using the statistical model that takes into account all the de-excitation processes occurring in photoexcited linear C_{10}^-. The results from the model for photoexcitation at different trapping times are depicted by the lines in Supplementary Figure 1. To calculate the neutralization rates, the initial internal energy population distributions at different trapping times were estimated using different vibrational temperatures. There is excellent agreement within the experimental error between the neutralization rates computed by the model and those measured experimentally.

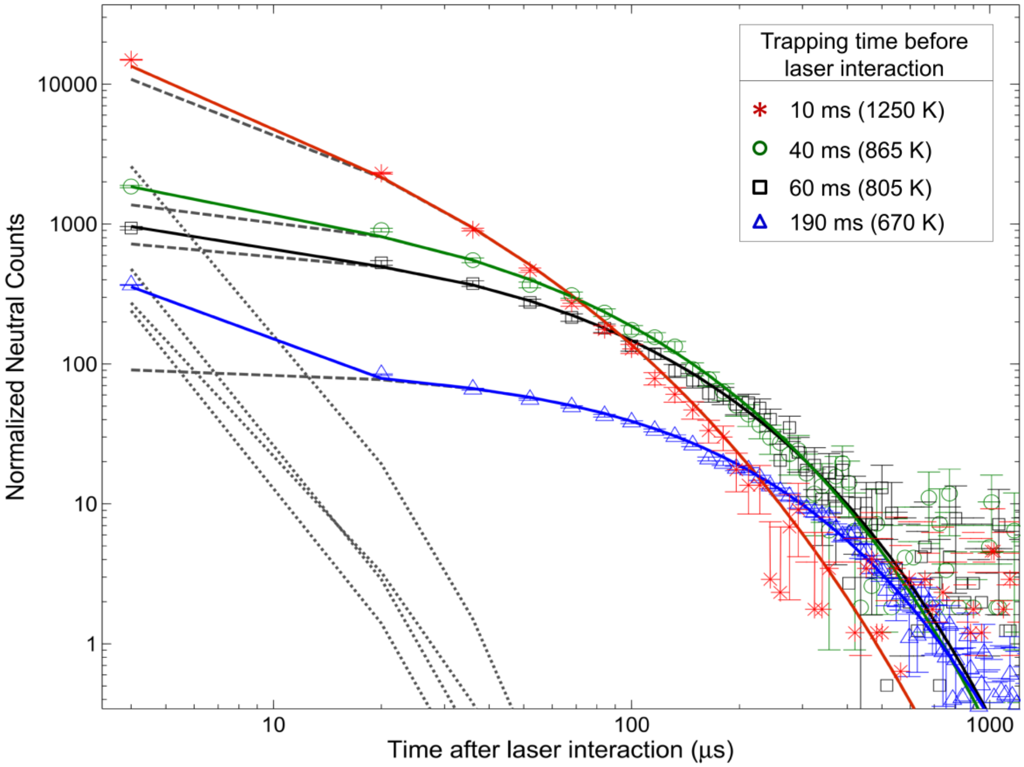

Supplementary Figure 1: Neutral counts as a function of time after laser interaction with C_{10}^{-} at different trapping times for photoexcitation energy of 2.85 eV. The symbols are the experimental data. The error bars are the standard deviation of the experimental data. The lines are from theoretical calculations using the model. The dashed lines denote contributions from one-photon excitations while the dotted lines are due to excitations from two photons. The solid lines represent the contribution from both and agree well with the experimental data. The vibrational temperatures at different trapping times as predicted by the model are shown in parentheses.

Supplementary Note 2: Estimation of prompt events upon photoexcitation of C_{10}^-

In our experiments, neutrals produced from cyclic C_{10}^- upon electron detachment should be prompt with respect to the laser interaction since the photon energies employed are much above its adiabatic electron affinity. Prompt is defined by our detection time resolution (50 ns) and the geometry of the experiment, as explained herein. A measure of the number of such promptly produced neutrals would indicate the amount of cyclic C_{10}^- present in the trapped ion beam. For ions trapped in a bent electrostatic ion beam trap (EIBT), unlike the delayed neutrals (i.e., neutrals produced after a delay with respect to photoexcitation), the neutrals produced due to prompt electron emission fly out of the linear section of our trap and would not be recorded by the detector located after the bent section exit mirror (see Fig. 1 of the main article). To estimate the number of neutrals produced due to prompt electron emission, we trapped the C_{10}^- ion beam in the bent EIBT and photoexcited the ions after 190 ms of trapping. The neutrals created as a result of photoexcitation that escape through the linear section exit mirror of the EIBT are detected by an microchannel plate (MCP) placed after the mirror[1]. The MCP detector used for this purpose has a central hole so that the laser pulse can be admitted into the EIBT. Due to this hole in the detector, there is a significant reduction in the number of neutrals that can be detected. Nevertheless, as this loss is identical for all neutrals regardless of their mechanism of creation, we are able to estimate the number of prompt neutrals relative to delayed neutrals. Neutrals produced after laser interaction are recorded as a function of time and are shown in Supplementary Figure 2 for photoexcitation energy of 2.85 eV.

The zero of the time axis of Supplementary Figure 2 is when the first laser-induced neutrals were detected. The neutral counts during the first ~3 μs (i.e., first 6 data points) are from ions that were travelling towards the detector between the entrance mirror and the deflector at the time of laser interaction. Prompt neutrals that are detected in our experiments come only from these ions. Delayed neutrals are also produced during this time period, so the neutral counts that we observe during the first 3 μs are a combination of prompt and delayed neutrals. Neutrals counts after 3 μs and up to ~7 μs are from ions that were travelling away from the detector at the time of laser interaction; any prompt neutrals produced from these ions will not be detected as they will exit the trap through the entrance mirror. Neutrals are detected from these ions when the ions are reflected by the entrance mirror and start travelling towards the detector. Thus, the neutral counts that are observed after 3 μs and up to ~7 μs are purely delayed neutrals. The counts after 7 μs may be from slow neutrals that are formed inside the entrance mirror or from stray particles.

To educe the number of prompt neutrals produced upon photoexcitation, we need to subtract the contribution of delayed neutrals from the counts during the first 3 μs. For such a short duration, the dependence of neutral counts on time can be approximated to be exponentially decaying. Thus the number of delayed neutrals produced in the first 3 μs can be estimated by fitting the delayed neutrals counts obtained after 3 μs and up to ~7 μs by an

exponentially decaying function and extrapolating it for shorter times (solid line in Supplementary Figure 2). Subtracting the number of delayed neutrals thus determined from the total neutral counts for the first 3 μs gives us an estimate of the number of prompt neutrals. Following this procedure, we estimate the number of such prompt neutrals to be less than 1% of the neutrals produced during the first 7 μs. Such a small fraction of neutrals formed due to prompt electron emission upon photoexcitation indicates the absence of a significant amount of cyclic C_{10}^- in the trapped ion beam. The estimation given here is not exact since additional corrections may influence this number; among them is the exact laser overlap, the exact ion beam profile and the detection efficiency, which may lead to a 30% systematic error[2]. It should be noted that in the rest of the figures we have used the ion oscillation period as our binning size.

It is worth mentioning that even if there was a significant amount of cyclic C_{10}^- in the trapped ion beam; it would not have contributed to the slow neutralization rate that we observed at these photon energies, which are much higher than its adiabatic electron affinity. Therefore, our analysis of the neutralization rate that we observe for long times after laser excitation is independent of the initial cyclic C_{10}^- population in the trapped ion beam for the photon energies at which the experiments were performed.

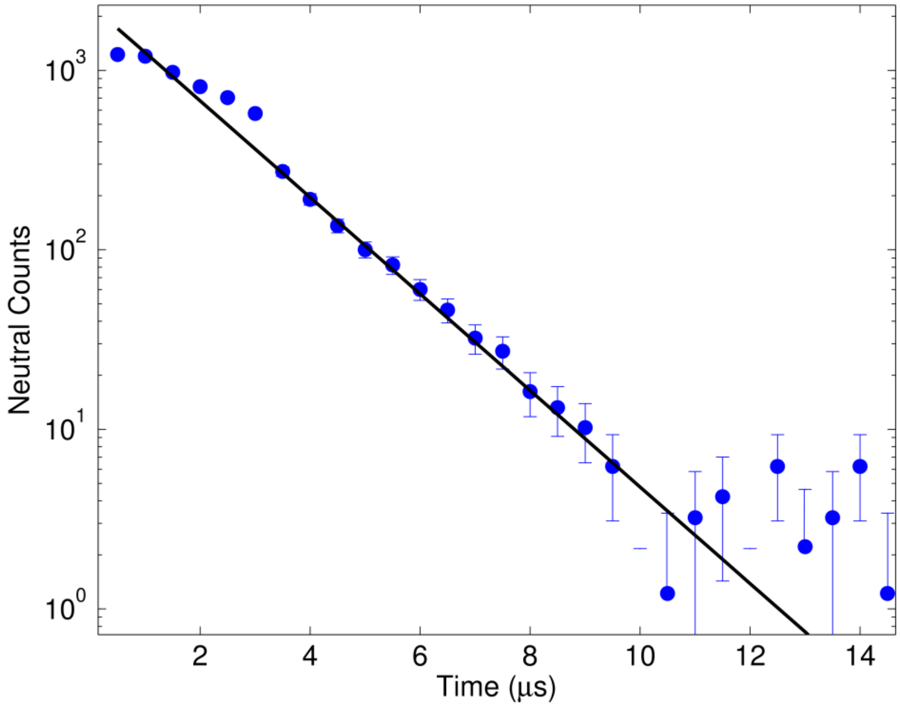

Supplementary Figure 2: Neutral counts (background subtracted) as a function of time due to photoexcitation of C_{10}^- by a 2.85 eV laser pulse after 190 ms of trapping. The zero of the time axis is when the first laser-induced neutrals were detected. The filled circles are the experimental data binned to 0.5 μs. The error bars are the standard deviation of the experimental data. The line is an exponential fit to the delayed neutral counts after 3 μs and up to ~7 μs (see text).

Supplementary References

1. Aviv, O. *et al.* A bent electrostatic ion beam trap for simultaneous measurements of fragmentation and ionization of cluster ions. Review of Scientific Instruments, **79**, 083110, (2008).

2. Kafle, B. *et al.* Electron detachment and fragmentation of laser-excited rotationally hot Al_4^-. Physical Review A, **92**, 052503, (2015).
